# Residue-Specific Incorporation of the Non-Canonical Amino Acid Norleucine Improves Lipase Activity on Synthetic Polyesters

**DOI:** 10.3389/fbioe.2022.769830

**Published:** 2022-01-26

**Authors:** Karolina Haernvall, Patrik Fladischer, Heidemarie Schoeffmann, Sabine Zitzenbacher, Tea Pavkov-Keller, Karl Gruber, Michael Schick, Motonori Yamamoto, Andreas Kuenkel, Doris Ribitsch, Georg M. Guebitz, Birgit Wiltschi

**Affiliations:** ^1^ Acib–Austrian Centre of Industrial Biotechnology, Graz, Austria; ^2^ Institute of Molecular Biotechnology, Graz University of Technology, Graz, Austria; ^3^ Institute of Molecular Biosciences, University of Graz, Graz, Austria; ^4^ BioTechMed-Graz, Graz, Austria; ^5^ Field of Excellence BioHealth—University of Graz, Graz, Austria; ^6^ BASF SE, Ludwigshafen am Rhein, Germany; ^7^ Institute for Environmental Biotechnology, University of Natural Resources and Life Sciences, Vienna, Austria

**Keywords:** polyester modification, enzyme hydrolysis, genetic code engineering, lipase, *Thermoanaerobacter thermohydrosulfuricus*, TTL, norleucine

## Abstract

Environmentally friendly functionalization and recycling processes for synthetic polymers have recently gained momentum, and enzymes play a central role in these procedures. However, natural enzymes must be engineered to accept synthetic polymers as substrates. To enhance the activity on synthetic polyesters, the canonical amino acid methionine in *Thermoanaerobacter thermohydrosulfuricus* lipase (TTL) was exchanged by the residue-specific incorporation method for the more hydrophobic non-canonical norleucine (Nle). Strutural modelling of TTL revealed that residues Met-114 and Met-142 are in close vicinity of the active site and their replacement by the norleucine could modulate the catalytic activity of the enzyme. Indeed, hydrolysis of the polyethylene terephthalate model substrate by the Nle variant resulted in significantly higher amounts of release products than the Met variant. A similar trend was observed for an ionic phthalic polyester containing a short alkyl diol (C5). Interestingly, a 50% increased activity was found for TTL [Nle] towards ionic phthalic polyesters containing different ether diols compared to the parent enzyme TTL [Met]. These findings clearly demonstrate the high potential of non-canonical amino acids for enzyme engineering.

**GRAPHICAL ABSTRACT:**
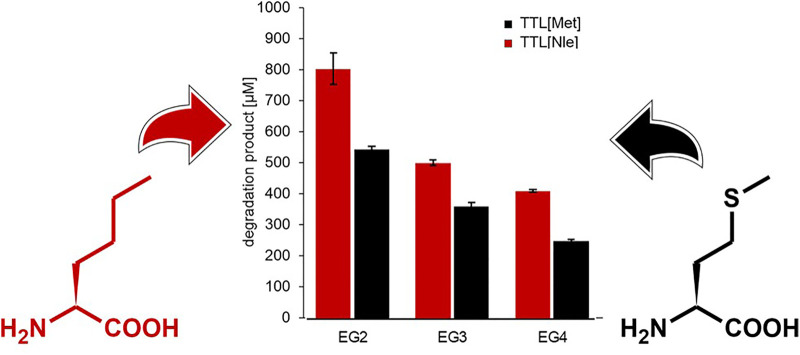


## Introduction

With the increasing utilization of synthetic polymers in today’s society, polymer surface functionalization and recycling processes have become enormously important. Surface modification is generally performed with harsh chemical methods, photo grafting, or energy-intensive plasma processes (Mozetič, 2019). These conventional methods are often toxic, expensive and adversely affect the mechanical properties of the polymer (Hetemi and Pinson, 2017). Enzymatic surface functionalization of polyesters has received increased attention as an environmentally friendlier and highly specific process (Pellis et al., 2015; Weinberger et al., 2017; Biundo et al., 2018). Limited enzymatic hydrolysis allows targeted surface functionalization while leaving the polymer bulk properties unaffected. Complete enzymatic hydrolysis of polyesters, on the other hand, allows specific recovery of the valuable building blocks for further synthesis especially from blends and composite materials which is otherwise quite challenging (Gamerith et al., 2017; Wei and Zimmermann, 2017). Enzymatic hydrolysis has so far been reported for various synthetic polymers such as poly-L-lactic acid (PLLA) (Pellis et al., 2015; Hajighasemi et al., 2016; Bonifer et al., 2019), polyethylene terephthalate (PET) (Herrero Acero et al., 2011; Sulaiman et al., 2012; Ribitsch et al., 2013; Kawai et al., 2014; Dimarogona et al., 2015; Barth et al., 2016; Yoshida et al., 2016; Kawabata et al., 2017), polybutylene adipate terephthalate (PBAT) (Biundo et al., 2016; Wallace et al., 2017) and polyurethanes (PU) (Acero et al., 2012; do Canto et al., 2019). To date, most enzymes used for polyester hydrolysis are esterases, lipases or cutinases from typical mesophilic or thermophilic compost microorganisms (Kleeberg et al., 2005; Herrero Acero et al., 2011; Ribitsch et al., 2011; Ribitsch et al., 2012a; Ribitsch et al., 2012b; Pellis et al., 2015). Only few enzymes were isolated from anaerobic (Biundo et al., 2016; Perz et al., 2016a; Perz et al., 2016b) and aquatic bacteria (Haernvall et al., 2017a; Haernvall et al., 2017b; Wallace et al., 2017; Haernvall et al., 2018).

Over the last years, several protein engineering strategies have been reported to tailor the enzymes for the non-natural polymeric substrates, thus improving the enzymatic hydrolysis of synthetic polymers. Different approaches have been applied to adapt the active site to the bulky polymers (Thumarat et al., 2012) and to reduce inhibition effects caused by release products (Roth et al., 2014). Besides, the adsorption/desorption of the enzymes on the polyester has been improved which turned out to be a crucial and rate-limiting step in enzymatic polymer hydrolysis (Herrero Acero et al., 2013; Ribitsch et al., 2013; Ribitsch et al., 2015). Today it is well known that temperatures higher than the glass transition temperature (Tg) increase the hydrolysis rates due to a higher flexibility of the polymer chains and therefore increased accessibility to enzymes (Kawai et al., 2019). For this reason, the thermostability of polymer hydrolysing enzymes has become a highly important issue and target for protein engineering (Shirke et al., 2016; Son et al., 2019).

The conventional protein engineering approaches use the 20 canonical amino acids (cAAs) prescribed by the genetic code (Steiner and Schwab, 2012). Still providing a very potential tool, conventional protein engineering is limited by the site-chain chemistry offered by the 20 cAAs. An alternative approach for generating more robust enzymes or altering physico-chemical properties is incorporation of non-canonical amino acids (ncAAs) (Hoesl and Budisa, 2012; Wiltschi et al., 2020). The diverse and unusual side chain chemistries (Dumas et al., 2015) makes them attractive building blocks for protein engineering (Link and Tirrell, 2003; Voloshchuk and Montclare, 2009; Zheng and Kwon, 2012; Wiltschi et al., 2020). Under tightly controlled conditions, many ncAAs can be incorporated into a target protein by exploiting the translational machinery of the host (Cowie and Cohen, 1957). This residue-specific incorporation (SPI) exploits the natural substrate tolerance of aminoacyl-tRNA synthetases (aaRS) for which various methods have been developed (Budisa, 2004; Hoesl et al., 2011; Ngo and Tirrell, 2011). The non-canonical amino acid norleucine (Nle) is an isosteric carba-analog of methionine (Met) that can be incorporated by the SPI method using a Met auxothrophic strain (Hoesl et al., 2011). The carbon atom of the methylene group of Nle is less electronegative than the corresponding sulfur atom of Met (2.48 vs 2.56) (Xie et al., 1995), resulting in a slightly increased hydrophobicity of the methylene group in comparison to the sulfur atom (Thomson et al., 1994). This subtle difference can exert a cumulative impact when several Met residues are globally exchanged for Nle. For instance, Budisa and co-workers successfully used Nle and other Met analogs to probe protein folding (Budisa et al., 1998) and control the aggregation behavior of prion protein (Wolschner et al., 2009). Nle is a comparably inexpensive ncAA. It can be produced in very good quantities by biosynthesis from glucose and the scalability of this bioprocess was previously demonstrated (Anderhuber et al., 2016).

In the past, several groups demonstrated that the incorporation of a palette of different ncAAs into target proteins could improve enzyme activity (Parsons et al., 1998; Cirino et al., 2003; Hoesl et al., 2011). NcAA incorporation can increase protein stability (Moroder and Budisa, 2010), especially under harsh conditions, such as tolerance to organic solvents (Deepankumar et al., 2014) or extreme pH values in combination with high temperatures (Votchitseva et al., 2006). For an overview of the engineering of enzymes with ncAAs, the interested reader is referred to (Wiltschi et al., 2020). A whole set of different ncAAs was successfully incorporated into the lipase from the anaerobic extreme thermophilic microorganisms *Thermoanaerobacter thermohydrosulfuricus* (TTL) by Budisa and coworkers (Acevedo-Rocha et al., 2013), demonstrating the positive effect on enzyme activity and stability in harsh conditions. TTL was first described by Royter and coworkers (Royter et al., 2009) as an enzyme with activity at a broad temperature (40°C—90°C) and pH range (pH 6.5–10). However, incorporation of ncAAs resulted not only in enhanced hydrolytic residual activity upon treatment with organic solvents by up to 450% and surfactants by up to 1630% (Hoesl et al., 2011), but has also reduced denaturation, alkylating and inhibition processes (Acevedo-Rocha et al., 2013).

In this study, we explored the incorporation of a ncAA into TTL to tune the physicochemical properties of the enzyme for enhanced polyester hydrolysis. As a proof of concept, the methionines (Met) of TTL were exchanged globally by its slightly more hydrophobic analog Nle. By the residue-specific incorporation of Nle into TTL, we aimed at altering the active site and/or the substrate-entrance channel to better fit the polymeric substrate. Synergistically, the enzyme surface was adapted to promote a better interaction/adhesion to the polymer. Two groups of polyester model substrates with various alkyl and ether diols were subjected to hydrolysis to evaluate the effect of Nle incorporation on polyester hydrolysis.

## Materials and Methods

### Chemicals and Substrates

Alkyl diols (1,5-pentanediol, 1,8-octanediol and 1,12-dodecanediol) and ether diols (diethylene glycol, triethylene glycol and tetraethylene glycol), terephthalic acid (TA) and 5-sulfoisophthalic acid (NaSIP) were purchased from Sigma-Aldrich (St. Louis, MO). Buffer components, bovine serum albumin (BSA) and methanol (HPLC grade) were also purchased from Sigma-Aldrich.

All standard chemicals used in this work were purchased from Sigma-Aldrich, Merck KGaA (Darmstadt, Germany) or Carl Roth (Karlsruhe, Germany), if not stated differently. Norleucine was obtained from IRIS Biotech GmbH (Marktredwitz, Germany). Enzymes for cloning and PCR were from Thermo Fisher Scientific (Waltham, MA). PCRs were performed using TaKaRa Ex Taq®High-Fidelity DNA polymerase (Clontech Laboratories, Inc., Mountain View, CA) or Dream Taq®DNA polymerase (Thermo Fisher Scientific). PCR primers were ordered from IDT Inc. (Coralville, IA) in standard desalted quality.

### Strains and Plasmids

*E. coli* B834 (DE3) (*E. coli* B F– ompT hsdSB(rB– mB–) dcm+ gal met λ(DE3); Merck KGaA) was the host for the SPI experiment and *E. coli* DH5α (*E. coli* K-12 F– endA1 supE44 thi-1 recA1 relA1 gyrA96 phoA φ80lacZ∆M15 ∆(lacZYA-argF)U169 hsdR17 (rK– mK+) λ–; Thermo Fisher Scientific) was used for cloning experiments and plasmid propagation. Transformation of *E. coli* was carried out by electroporation as described by Seidman et al. (2001). pTTL was constructed according to Anderhuber et al. (2016). Briefly, the coding sequence of the lipase from *T. thermohydrosulfuricus* was PCR-amplified from synthetic DNA (IDT Inc.) using primers BPp244 and BPp245 (Anderhuber et al., 2016). The PCR fragment was inserted into pQE80L (Qiagen, Hilden, Germany) cut with EcoRI and HindIII by Gibson isothermal assembly (Gibson et al., 2009).

### Enzyme Expression and Purification

The SPI experiment was performed as described previously (Anderhuber et al., 2016). Briefly, M9 minimal medium (M9 MM) containing M9 salt buffer (48 mM Na_2_HPO_4_, 22 mM KH_2_PO_4_, 9 mM NaCl and 19 mM NH_4_Cl) was used supplemented with 1 mM MgSO_4_, 7 µM CaCl_2_, 1 mg/L thiamine and trace elements (9 µM FeSO_4_, 3.5 µM MnSO_4_, 2.5 µM AlCl_3_, 2 μM CoCl_2_, 0.4 µM ZnSO_4_, 0.5 µM Na_2_MoO_4_, 0.4 µM CuCl_2_ and 0.5 µM H_3_BO_4_). 100 mg/L ampicillin was added for plasmid maintenance. 20 mM glucose was provided as the carbon source. For Met depletion at OD_600_∼ 3, 3.5 g/L yeast extract (Carl Roth) were supplemented in the medium. Cells were grown in baffled shake flasks at 37°C with vigorous shaking. After depletion of Met, as indicated by growth arrest, the cultures were supplemented with 1 mM of either Met (cAA, parent TTL [Met]) or Nle (Nle, synthetic TTL [Nle]). Gene expression was induced by adding IPTG (VWR International, Vienna, Austria) to a final concentration of 0.5 mM and performed with vigorous shaking for 4 h at 30°C. Cells were harvested at 4,000 g for 20 min at 4°C.

Cell pellets were resuspended in 30 ml Ni-NTA Lysis Buffer (50 mM NaH_2_PO_4_, 300 mM NaCl, 10 mM imidazole, pH 7.4) and incubated for 30 min on ice. Cells were disrupted by sonication on ice (Branson Sonifier 250, Emerson Electric, St. Louis, MO). The sonication was performed for 6 min with the following settings: duty cycle: 70%, output: 7-8, sonication tip Φ∼1 cm. After centrifugation for 45 min at 40,000 x g and 4°C the lysates were filtered through 0.2 µm syringe filters and purified on Ni-NTA sepharose according to the manufacturer’s protocol (IBA GmbH, Goettingen, Germany). Finally, the buffer was exchanged for 100 mM Tris-HCl pH 7.0 with PD-10 columns (GE Healthcare, Chicago, IL).

### Protein Quantification

The Bradford based Bio-Rad Protein Assay (Bio-Rad Laboratories GmbH, Munich, Germany) with bovine serum albumin as standard was used to determine protein concentrations of purified enzymes. The protein assay was performed according to the manufacturer’s instruction.

### Intact Protein Mass Determination

The protein solutions were desalted using Amicon Ultra 0.5 ml centrifugal filter units (Millipore, Billerica, MA). A final protein concentration of 30 pmol/µL was obtained with water containing 5% acetonitrile and 0.1% trifluoroacetic acid. The separation of possible protein variants was carried out on a capillary HPLC system (1200 Agilent, Agilent Technologies) using a PepSwift RP monolithic column (50 × 0.5 mm, Thermo Fisher Scientific) at a flow rate of 20 µL/min and a column temperature of 60°C. The gradient of solution A (water +0.05% TFA) and B (ACN +0.05% TFA) was performed as follows: 10% B for 5 min, 10%–100% B for 50 min, 100%–10% B for 1 min, 10% B for 15 min. The injection volume was 5 µL. The Thermo LTQ-FT mass spectrometer (Thermo Fisher Scientific) was operated with an ESI source in positive mode with the following settings: mass range: 300–2000 m/z, resolution 400000, 500 ms injection time, 1 microscan, source voltage 5 kV, capillary voltage 35 V, sheath gas flow 15. The protein mass spectra were deconvoluted by the Thermo Fisher Scientific software Protein Deconvolution 2.0, using the Xtract algorithm. The following main parameters were applied: charge carrier, H+; m/z range, minimal 800 to maximal 2000; minimal detected charge state, 4; s/n threshold, 5; relative abundance threshold, 20%. Trace amounts of unlabeled species might be present but fall below the detection limit of the mass spectrometry method (2–5%).

### Structure Modeling

The structure of the TTL was modeled using the CATALOphore platform of Innophore GmbH (www.innophore.com) employing the program Yasara version 20.4.24.L.64 (www.yasara.org). The model is based on the structure of feruloyl esterase (Est1E) from *Butyrivibrio proteoclasticus* (PDB 2WTM) (Goldstone et al., 2010) as template, which shares 32% sequence identity and 50% sequence similarity with TTL. The active site cavity in the vicinity of the catalytic triad Ser-113, His-233 and Asp-203 was calculated using the program CavMan (Innophore GmbH, www.innophore.com) employing the LIGSITE algorithm (Hendlich et al., 1997). For the analysis of the hydrophobicity of the cavity, the hydrophobicity module of the program VASCo (Steinkellner et al., 2009) as implemented in CavMan was used.

### Polyester Synthesis and Characterization

The oligomeric model substrate bis-(benzoyloxyethyl) terephthalate (3PET) was synthesized as previously reported (Heumann et al., 2009). Other polyesters were synthesized in a two-step process and characterized as described by Haernvall et al. (2017b).

### Hydrolysis of Oligomeric and Polymeric Materials

Model Substrates Polyester powders (10 mg/ml) were incubated in 1 ml of 100 mM Tris-HCl pH 7.0 and in the presence of 1 μM enzyme. The reaction mixture was shaken for 3 h at 70°C and 100 rpm (Infors HT Multitron, Infors AG, Bottmingen, Switzerland). In parallel, enzymes and polymers were incubated in pure buffer as blank reactions. All experiments were run in triplicates. Enzymes were precipitated by addition of ice-cold methanol (1:1 v/v), acidified with 0.1 M HCl to pH 4 and sedimented in a tabletop centrifuge (15 min, 0°C, 14,000 rpm; Hermle Labortechnik GmbH, Wehingen, Germany). Supernatants were used for HPLC analysis.

### Determination of Hydrolysis Products

After hydrolysis, samples were analyzed by HPLC-UV on a system consisting of a Dionex UltiMate 181 3000 Pump (Dionex Cooperation, Sunnyvale, United States), a Dionex ASI-100 automated sample injector, a Dionex UltiMate 3000 column compartment and a Dionex UVD 340 U photodiode array detector. The hydrolysis products were separated by a reversed-phase column, [XTerra® RP18, (3.5 μm, 3.0 mm × 150 mm)] (Waters Corporation, Milford, United States) using a non-linear gradient where eluent A consisted of water, eluent B of methanol and eluent C of 0.01 N sulfuric acid. The separation was achieved by a non-linear gradient increased from 15% A to 40% A from 13 to 30 min, followed by an increase to 90% A during 5 min which was kept for 10 min to then be re-established to initial conditions within 1 min and equilibrated for 20 min. The injection volume was 5 μL, and the flow rate was 0.4 ml/min. The column compartment had a constant temperature of 40°C. The expected release products TA and NaSIP were detected *via* UV/VIS spectroscopy, and the release products were qualified and quantified based on calibration curves.

### Enzyme Adsorption on PET

Enzyme adsorption on PET film was monitored as previously described (Ribitsch et al., 2013). Briefly, 0.5 × 1 cm^2^ PET films were washed at 50°C in three consecutive steps of 30 min each with 1.5 ml each of 0.5% (v/v) Triton X-100, 100 mM Na_2_CO_3_ and deionized water. The washed membranes were incubated in 1 ml of 0.6 mg/ml enzyme solution for 2 h at 30°C. The films were washed twice by dipping into 1.5 ml TBST (25 mM Tris, 0.15 M NaCl, pH 7.6, 0.05% (v/v) Tween®-20) at room temperature. Then, the films were incubated with HisProbe (1:2500 dilution in TBST; SuperSignal West HisProbe Kit; Thermo Fisher Scientific, Waltham, MA) for 40 min at room temperature with shaking. The films were washed three times by dipping into 1.5 ml TBST as described above and were developed by incubating in 600 µL of SuperSignal West Pico Substrate Working Solution for 5 min. Chemiluminescence signals were detected using G:box Chemi HR16 and GeneSnap image acquisition software Version 7.05.01 (Syngene, Cambridge, United Kingdom) and quantified with Colorlite sph850 (Colorlite:Inovative color measurements; Germany).

## Results and Discussion

**FIGURE 1:**
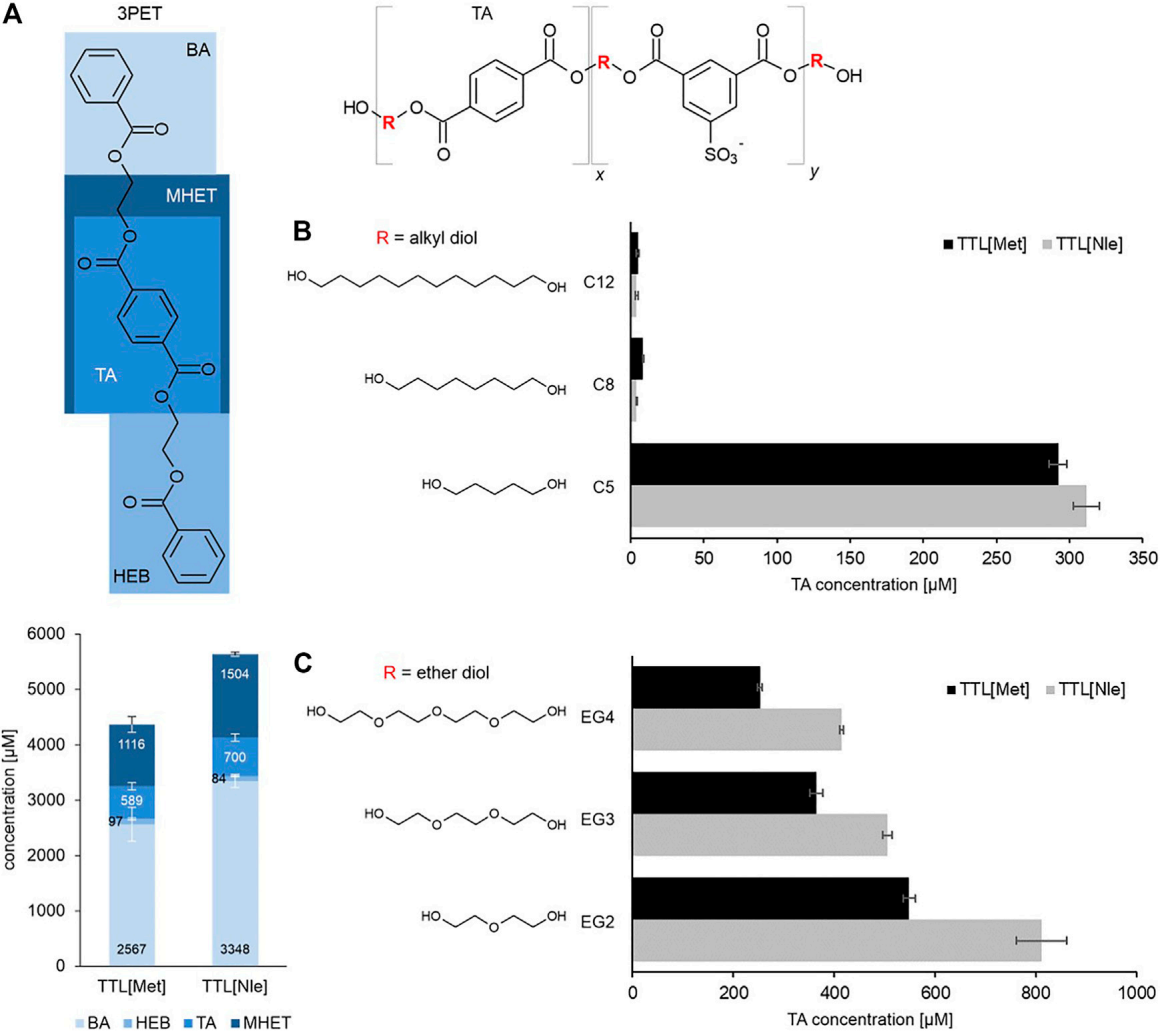
Hydrolysis of polyester model substrates **(A)** Hydrolysis of the oligomeric model substrate bis-(benzoyloxyethyl) terephthalate (3PET) with 0.6 µM TTL [Nle] and TTL [Met] at pH 7.0. Total released molecules after 24 h of incubation at 70°C. The release products BA, benzoic acid; HEB, hydroxyethylbenzoate; TA, terephthalic acid and MHET, Mono-(2-hydroxyethyl)terephthalic acid were quantified by HPLC analysis. Each bar represents the average of three independent samples; error bars indicate the standard deviation. Hydrolysis of polyesters consisting of 70 mol% terephthalic acid (Ta) and 30 mol% 5-sulfoisophthalic acid (NaSIP) and the respective alkyl and ether diols with different chain lengths of **(B)** alkyl diols (C5, C8, and C12) and **(C)** ether diols (EG2, EG3, and EG4). Results obtained from hydrolysis with 1 μM TTL [Nle] and TTL [Met] at 70°C represented as the release of terephthalic acid after 24 h of incubation. Each bar represents the average of three independent samples; error bars indicate the standard deviation. Two-tailed p-values from unpaired t-tests were 0.047 (C5), <0.001 (C8), 0.40 (C12), 0.001 (EG2), <0.001 (EG3) and <0.001 (EG4). Abbreviations: 1,5-pentanediol (C5), 1,8-octanediol (C8), 1,12-dodecanediol (C12) and ether diols with different chain lengths: EG1: ethylene glycol, EG2: diethylene glycol, EG3: triethylene glycol and EG4: tetraethylene glycol.

The increasing interest in enzymatic polymer functionalization and enzymatic polymer recycling processes has resulted in various proposed strategies to improve the enzymatic hydrolysis of synthetic polymers. Here, in order to evaluate a novel approach, the classical protein engineering strategies were expanded to also include ncAAs. To assess the effects on enzymatic polymer hydrolysis, a lipase from an anaerobic extreme thermophilic microorganism was modified by exchanging the cAA methionine with the slightly more hydrophobic ncAA norleucine. The effects were evaluated towards oligomeric and polymeric polyester model substrates with systematically varied compositions to enable a mechanistic study. Therefore, a set of structurally different polyester substrates was used (Figure 1). The water-insoluble PET model substrate bis(benzoyloxyethyl) terephthalate (3PET, Figure 1A) has been widely used in the past for the detection of PET hydrolysing enzymes such as cutinases (Herrero Acero et al., 2011; Ribitsch et al., 2011; Kawai et al., 2019).

Only few enzymes were identified so far that show an ability to hydrolyze ionic phthalic acid based polyesters (Haernvall et al., 2017a). Ionic phthalic polyesters are found in many products in our daily life, such as household products. In this study, we used model polyesters consisting of the aromatic terephthalic acid (TA) and the ionic aromatic 5-sulfoisophthalic acid (NaSIP), which were linked by altering alkyl and ether diols. Two groups of ionic phthalic polyesters were used to evaluate the effect of chain length and hydrophilicity/hydrophobicity of polyesters influencing the hydrolysis. The first group of polyesters contained alkyl diols (1,5-pentanediol (C5), 1,8-octanediol (C8) and 1,12-dodecanediol (C12) (Figure 1B). The second group of polyesters contained ether diols (diethylene glycol (EG2), triethylene glycol (EG3) and tetraethylene glycol (EG4)) (Figure 1C), with systematically varied chain lengths. The ratio of TA and NaSIP was kept constant (70:30 mol%).

### Structure Modelling of TTL

**FIGURE 2:**
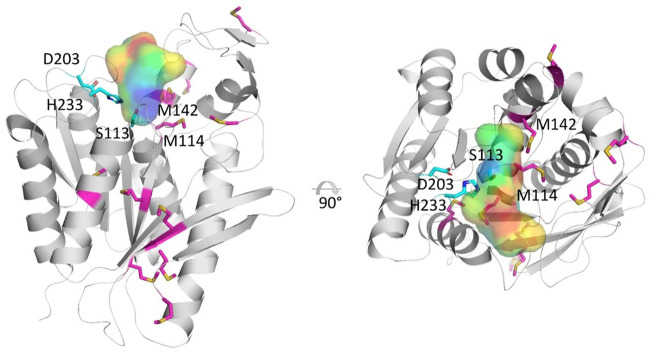
Cartoon representation of the modelled structure of the lipase from *Thermoanaerobacter thermohydrosulfuricus* in two, 90°-separated orientations. Amino acids forming the catalytic triad (Ser-113, His-233 and Asp-203) are shown in a cyan stick representation, while methionine residues are shown in magenta. The active site cavity is represented as a semi-transparent surface colored according to hydrophobicity, ranging from blue (hydrophilic) to red (hydrophobic). The figure was generated using the program PyMOL (www.pymol.org).

The modelled structure of the lipase from *Thermoanaerobacter thermohydrosulfuricus* (TTL) exhibits a typical α/β-hydrolase fold with a central eight-stranded, mostly parallel β-sheet flanked by six α-helices (Figure 2). A catalytic triad is formed by the amino acid residues Ser-113, His-233 and Asp-203, with the serine being embedded in a GLSMGG sequence motif. The oxyanion hole is built up by the mainchain amide groups of Phe-37 and Met-114. The active site cavity is located at the upper end of the α/β-hydrolase core and delimited by a small cap domain consisting of residues 140 to 180. The TTL-sequence contains a total of 11 methionine residues, of which Met-114 and Met-142 are in close vicinity to the active site residue (Figure 2). It is conceivable that the replacement of those two methionine residues by norleucine modulates the catalytic activity of the enzyme most likely by altering its substrate binding properties. Most of the remaining methionine residues are part of the hydrophobic core of the protein (Figure 2). Amino acid replacements in this region could change the dynamic properties of the enzyme and its stability. Indeed, Hoesl et al. (2011) observed that the quantitative replacement of the Met residues in TTL resulted in a variant enzyme that was highly active without thermal activation while the parent enzyme containing exclusively Met residues was nearly inactive unless heat activated. Hoesl et al. hypothesized that the global replacement of Met by Nle would increase the hydrophobicity around the substrate binding site, which could enhance the accessibility of the substrate into TTL [Nle] in aqueous solution.

### TTL Expression, Purification and Characterization

TTL was expressed in *E. coli* and purified from cleared cell lysates over a C-terminal 6xHisTag by affinity chromatography. Expression in the presence of the canonical Met yielded a defined band migrating at the 29.2 kDa molecular weight marker band as calculated for the theoretical molecular weight of TTL (Supplementary Figure S1). In contrast, the lipase expressed in the presence of Nle appeared as a blurred band at slightly lower molecular weight. In agreement with a previous report (Hoesl et al., 2011), the Nle variant exhibited accelerated electrophoretic mobility compared to the Met variant. The altered migration behavior was a first indication for the successful incorporation of Nle into the lipase. Typically, 20 mg/ml TTL [Met] and 10 mg/ml TTL [Nle)] were purified from 3 to 4 g of cell pellet obtained from cultures in shake flasks.

To analyze the incorporation efficiency of Nle, Met and Nle variants were subjected to intact protein mass analysis (Supplementary Figure S2). In the mass spectrum of TTL [Nle] the protein species with all 11 Met residues exchanged for Nle was the most prominent peak. A minor peak representing lipase with 10 Met residues exchanged for Nle was detected as well. This is in good agreement with previously published results (Hoesl et al., 2011).

### Hydrolysis of Oligomeric and Polymeric Model Substrates

The effect of the incorporating Nle into TTL on polymer degradation was assessed in a first step using the oligomeric model substrate 3PET. Interestingly, the global Met→Nle substitution in TTL had a positive impact on the hydrolysis as indicated by a ∼30 % higher amounts of hydrolysis products than to the parent enzyme (Figure 1A). Nevertheless, the pattern of the released molecules was similar for the two enzymes TTL (Met) and TTL (Nle). Benzoic acid (BA) was released in the highest concentration, followed by mono-(2-hydroxyethyl)terephthalic acid (MHET), terephthalic acid (TA) and hydroxyethylbenzoate (HEB), respectively. The total amount of released molecules for the hydrolysis of the oligomer model substrates was in the same range as previously reported for other enzymes, such as esterases from *Clostridium botulinum* (Perz et al., 2016a), a lipase from *Thermomyces lanuginosus* (Eberl et al., 2009), or cutinases from *Fusarium solani pisi* as well as from various *Thermobifida* species (Herrero Acero et al., 2011; Ribitsch et al., 2012b).

To further evaluate the impact of norleucine incorporation on polyester hydrolysis and to obtain a more detailed mechanistic insights into the hydrolysis behavior of TTL [Nle], a set of structurally different ionic phthalic acid polyesters were investigated. In a first step, TTL [Nle] was incubated with polymeric model substrates containing alkyl diols of different chain lengths, C5, C8, and C12 (Figure 1B). TTL [Nle] and the parent enzyme TTL [Met] were both active towards C5 while only a very low activity was detected towards C8 and C12 as indicated by the release of terephthalic acid. TTL [Nle] showed around 5% more activity towards C5 compared to TTL [Met]. However, enzymatic hydrolysis of polymeric substances is influenced by several parameters, such as water solubility of the substrates, crystallinity, molecular weight, glass transition temperature (Tg) and therefore difficult to predict (Chamas et al., 2020). As described recently (Haernvall et al., 2017b), polyesters C8 and C12 have lower water solubility, higher hydrophobicity and a higher crystallinity of 4 and 12%, respectively, than C5 with crystallinity below 1%, which may have influenced the hydrolysis. Low water solubility, high hydrophobicity (Okada et al., 1997) and crystallinity have previously been reported to have a negative impact on the enzymatic hydrolysis of polymers (Donelli et al., 2010). However, the glass transition temperature of the polymeric model substrates is decreasing with increasing chain length which would have been expected to have a positive impact on the enzymatic hydrolysis due to the higher flexibility of the polymer chains (Marten et al., 2003).

In a next step, TTL [Nle] and the parent enzyme TTL [Met] were incubated with the ether diol containing model substrates, EG2, EG3, EG4 (Figure 1C). Surprisingly, an up to 40% increased activity was found for TTL [Nle] towards all three polyesters (EG2, EG3, and EG4) when compared to the parent enzyme TTL [Met]. The highest activity was again detected on the shortest diol chain length, namely EG2, decreasing with increasing chain length. It can also be noted that an overall higher activity was detected for both enzymes towards all three ether diol containing substrates when compared to the alkyl diol analogs. This can be a consequence of the increased water solubility of the ether diol containing polymers and the increased hydrophilicity. It has previously been shown that increased water solubility and increased hydrophilicity is enhancing enzymatic hydrolysis. It has even been suggested as a parameter to tune polymeric biodegradation (Gigli et al., 2013).

In none of the cases, NaSIP was detected after hydrolysis, indicating that TTL [Met] and TTL [Nle] have a limited capacity to cleave ester bonds in close vicinity to the ionic monomer NaSIP. These results are in accordance with previously reported data for cutinase A and an arylesterase from *Pseudomonas pseudoalcaligenes* and a lipase from *Pseudomonas pelagia* (Haernvall et al., 2017a; Haernvall et al., 2017b).

**FIGURE 3:**
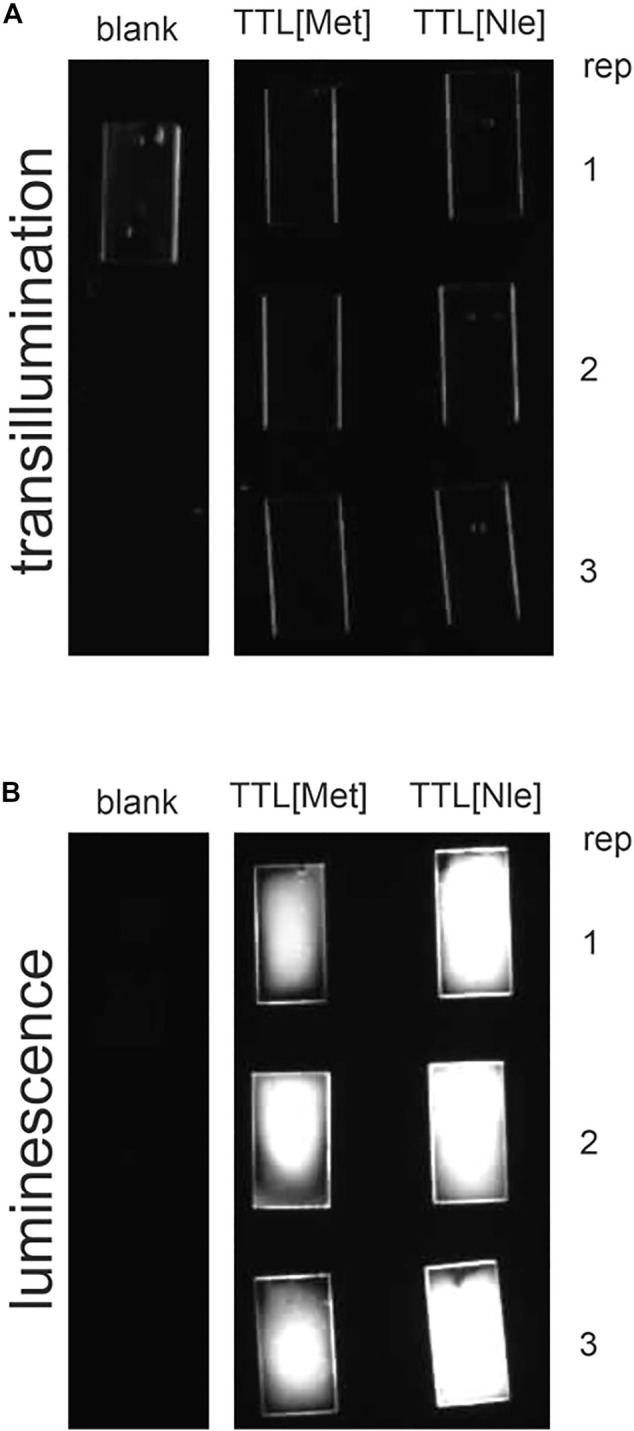
Adhesion of TTL [Met] and TTL [Nle] on PET film. Transillumination **(A)**; luminescence after detection of the hexahistidine-tag using HisProbe **(B)**.

We analyzed the adsorption of TTL [Met] and TTL [Nle] on PET films as described in the methods section. Adsorbed enzymes were detected by binding of horseradish peroxidase labeled HisProbe to the hexahistidine-fusion tag and chemiluminescence detection. TTL [Nle] adsorbed reproducibly better to the PET films than the parent protein (Figure 3). This result supports our notion that Nle improves the interaction of TTL with the polymer surface although a different polyester had been used. Both, TTL [Met] and TTL [Nle] survived well the incubation with PET because their esterase activities after the incubation, e.g., with PET, was not lower than before (Supplementary Information, Supplementary Figure S3). Our finding corroborates the extraordinary stability of TTL and its Nle reported previously (Hoesl et al., 2011).

## Conclusion

Recently, ncAAs have become a valuable asset for protein engineering, e.g., to introduce non-natural chemical functionalities into enzymes. In this study we have shown for the first time that incorporation of the ncAA Nle into a lipase from *Thermoanaerobacter thermohydrosulfuricus* has a positive impact on the enzymatic hydrolysis of synthetic polyesters. Nle, the carba-analog of Met, is less polar and more hydrophobic than Met while the structures are virtually identical. The global replacement of Met by Nle can tune the hydropathy of a protein in a subtle way that is difficult to attain by exchanging Met with a hydrophobic canonical amino acid. Nle lacks the sulfur atom which is replaced by a methylene group. Due to the lack of the sulfur atom, Nle cannot form sulfoxides protecting the enzymes from oxidative stress and is also claimed to prevent protein aggregation and chemical degradation. Our structural model of TTL predicts Met-114 and Met-142 to be ocated in close vicinity of the catalytic triad and the active site cavity and two other methionines, Met-147 and Met-158, to be part of the lid. Previously, Budisa et al. (Acevedo-Rocha et al., 2013) hypothesized that replacing methionine residues in the lid could have retained it in an open conformation. This together with the replacement of Met-114 and Met-142 near the catalytic triad might have modified the environment in the substrate entry tunnel and the catalytic site such that the hydrolysis of the hydrophobic synthetic polymers was enhanced. Further studies reveal whether a similar enhancing effect can be elicited in other polyester hydrolyzing enzymes that accommodate Met residues in their substrate entry tunnels and/or active sites. If this is the case, Nle–and eventually also other ncAAs–could complement the existing tools for tuning and improving the activity of enzymes on polymers.

## Data Availability

The original contributions presented in the study are included in the article/Supplementary Material, further inquiries can be directed to the corresponding author.

## References

[B1] Acero E. H. Ribitsch D. Rodriguez R. D. Dellacher A. Zitzenbacher S. Marold A. (2012). Two-step Enzymatic Functionalisation of Polyamide with Phenolics. J. Mol. Catal. B: Enzym. 79, 54–60. 10.1016/j.molcatb.2012.03.019

[B2] Acevedo-Rocha C. G. Hoesl M. G. Nehring S. Royter M. Wolschner C. Wiltschi B. (2013). Non-canonical Amino Acids as a Useful Synthetic Biological Tool for Lipase-Catalysed Reactions in Hostile Environments. Catal. Sci. Technol. 3 (5), 1198–1201. 10.1039/c3cy20712a

[B3] Anderhuber N. Fladischer P. Gruber-Khadjawi M. Mairhofer J. Striedner G. Wiltschi B. (2016). High-level Biosynthesis of Norleucine in *E. coli* for the Economic Labeling of Proteins. J. Biotechnol. 235, 100–111. 10.1016/J.JBIOTEC.2016.04.033 27107466

[B4] Barth M. Honak A. Oeser T. Wei R. Belisário-Ferrari M. R. Then J. (2016). A Dual Enzyme System Composed of a Polyester Hydrolase and a Carboxylesterase Enhances the Biocatalytic Degradation of Polyethylene Terephthalate Films. Biotechnol. J. 11 (8), 1082–1087. 10.1002/BIOT.201600008 27214855

[B5] Biundo A. Hromic A. Pavkov-Keller T. Gruber K. Quartinello F. Haernvall K. (2016). Characterization of a Poly(butylene Adipate-Co-Terephthalate)-Hydrolyzing Lipase from *Pelosinus Fermentans* . Appl. Microbiol. Biotechnol. 100 (4), 1753–1764. 10.1007/s00253-015-7031-1 26490551

[B6] Biundo A. Ribitsch D. Guebitz G. M. (2018). Surface Engineering of Polyester-Degrading Enzymes to Improve Efficiency and Tune Specificity. Appl. Microbiol. Biotechnol. 102 (Issue 8), 3551–3559. Springer Verlag. 10.1007/s00253-018-8850-7 29511846

[B7] Bonifer K. S. Wen X. Hasim S. Phillips E. K. Dunlap R. N. Gann E. R. (2019). *Bacillus Pumilus* B12 Degrades Polylactic Acid and Degradation Is Affected by Changing Nutrient Conditions. Front. Microbiol. 10, 2548. 10.3389/FMICB.2019.02548/BIBTEX 31824441 PMC6882738

[B8] Budisa N. Huber R. Golbik R. Minks C. Weyher E. Moroder L. (1998). Atomic Mutations in Annexin V. Thermodynamic Studies of Isomorphous Protein Variants. Eur. J. Biochem. 253 (1), 1–9. 10.1046/J.1432-1327.1998.2530001.X 9578454

[B9] Budisa N. (2004). Prolegomena to Future Experimental Efforts on Genetic Code Engineering by Expanding its Amino Acid Repertoire. Angew. Chem. Int. Ed. 43 (47), 6426–6463. Angewandte Chemie - International Edition. 10.1002/anie.200300646 15578784

[B10] Chamas A. Moon H. Zheng J. Qiu Y. Tabassum T. Jang J. H. (2020). Degradation Rates of Plastics in the Environment. ACS Sustain. Chem. Eng. 8 (9), 3494–3511. 10.1021/acssuschemeng.9b06635

[B11] Cirino P. C. Tang Y. Takahashi K. Tirrell D. A. Arnold F. H. (2003). Global Incorporation of Norleucine in Place of Methionine in Cytochrome P450 BM-3 Heme Domain Increases Peroxygenase Activity. Biotechnol. Bioeng. 83 (6), 729–734. 10.1002/bit.10718 12889037

[B12] Cowie D. B. Cohen G. N. (1957). Biosynthesis by *Escherichia coli* of Active Altered Proteins Containing Selenium Instead of Sulfur. Biochim. Biophys. Acta 26 (2), 252–261. 10.1016/0006-3002(57)90003-3 13499359

[B13] Deepankumar K. Shon M. Nadarajan S. P. Shin G. Mathew S. Ayyadurai N. (2014). Enhancing Thermostability and Organic Solvent Tolerance of ω-Transaminase through Global Incorporation of Fluorotyrosine. Adv. Synth. Catal. 356 (5), 993–998. 10.1002/adsc.201300706

[B14] Dimarogona M. Nikolaivits E. Kanelli M. Christakopoulos P. Sandgren M. Topakas E. (2015). Structural and Functional Studies of a *Fusarium Oxysporum* Cutinase with Polyethylene Terephthalate Modification Potential. Biochim. Biophys. Acta (Bba) - Gen. Subjects 1850 (11), 2308–2317. 10.1016/J.BBAGEN.2015.08.009 26291558

[B15] do Canto V. P. Thompson C. E. Netz P. A. (2019). Polyurethanases: Three-Dimensional Structures and Molecular Dynamics Simulations of Enzymes that Degrade Polyurethane. J. Mol. Graphics Model. 89, 82–95. 10.1016/J.JMGM.2019.03.001 30877946

[B16] Donelli I. Freddi G. Nierstrasz V. A. Taddei P. (2010). Surface Structure and Properties of Poly-(ethylene Terephthalate) Hydrolyzed by Alkali and Cutinase. Polym. Degrad. Stab. 95 (9), 1542–1550. 10.1016/j.polymdegradstab.2010.06.011

[B17] Dumas A. Lercher L. Spicer C. D. Davis B. G. (2015). Designing Logical Codon Reassignment - Expanding the Chemistry in Biology. Chem. Sci. 6 (1), 50–69. 10.1039/c4sc01534g 28553457 PMC5424465

[B18] Eberl A. Heumann S. Brückner T. Araujo R. Cavaco-Paulo A. Kaufmann F. (2009). Enzymatic Surface Hydrolysis of Poly(ethylene Terephthalate) and Bis(benzoyloxyethyl) Terephthalate by Lipase and Cutinase in the Presence of Surface Active Molecules. J. Biotechnol. 143 (3), 207–212. 10.1016/j.jbiotec.2009.07.008 19616594

[B19] Gamerith C. Zartl B. Pellis A. Guillamot F. Marty A. Acero E. H. (2017). Enzymatic Recovery of Polyester Building Blocks from Polymer Blends. Process Biochem. 59, 58–64. 10.1016/j.procbio.2017.01.004

[B20] Gibson D. G. Young L. Chuang R.-Y. Venter J. C. Hutchison C. A. Smith H. O. (2009). Enzymatic Assembly of DNA Molecules up to Several Hundred Kilobases. Nat. Methods 6 (5), 343–345. 10.1038/nmeth.1318 19363495

[B21] Gigli M. Lotti N. Gazzano M. Finelli L. Munari A. (2013). Synthesis and Characterization of Novel Poly(butylene Succinate)-Based Copolyesters Designed as Potential Candidates for Soft Tissue Engineering. Polym. Eng. Sci. 53 (3), 491–501. 10.1002/pen.23289

[B22] Goldstone D. C. Villas-Bôas S. G. Till M. Kelly W. J. Attwood G. T. Arcus V. L. (2010). Structural and Functional Characterization of a Promiscuous Feruloyl Esterase (Est1E) from the Rumen Bacterium Butyrivibrio Proteoclasticus. Proteins 78 (6), 1457–1469. 10.1002/prot.22662 20058325

[B23] Haernvall K. Zitzenbacher S. Wallig K. Yamamoto M. Schick M. B. Ribitsch D. (2017a). Hydrolysis of Ionic Phthalic Acid Based Polyesters by Wastewater Microorganisms and Their Enzymes. Environ. Sci. Technol. 51 (8), 4596–4605. 10.1021/acs.est.7b00062 28345898

[B24] Haernvall K. Zitzenbacher S. Yamamoto M. Schick M. B. Ribitsch D. Guebitz G. M. (2017b). A New Arylesterase from *Pseudomonas pseudoalcaligenes* Can Hydrolyze Ionic Phthalic Polyesters. J. Biotechnol. 257, 70–77. 10.1016/j.jbiotec.2017.01.012 28237250

[B25] Haernvall K. Zitzenbacher S. Biundo A. Yamamoto M. Schick M. B. Ribitsch D. (2018). Enzymes as Enhancers for the Biodegradation of Synthetic Polymers in Wastewater. ChemBioChem 19 (4), 317–325. 10.1002/cbic.201700364 29119717

[B26] Hajighasemi M. Nocek B. P. Tchigvintsev A. Brown G. Flick R. Xu X. (2016). Biochemical and Structural Insights into Enzymatic Depolymerization of Polylactic Acid and Other Polyesters by Microbial Carboxylesterases. Biomacromolecules 17 (6), 2027–2039. 10.1021/ACS.BIOMAC.6B00223 27087107 PMC6886529

[B27] Hendlich M. Rippmann F. Barnickel G. (1997). LIGSITE: Automatic and Efficient Detection of Potential Small Molecule-Binding Sites in Proteins. J. Mol. Graphics Model. 15 (6), 359–363. 10.1016/S1093-3263(98)00002-3 9704298

[B28] Herrero Acero E. Ribitsch D. Steinkellner G. Gruber K. Greimel K. Eiteljoerg I. (2011). Enzymatic Surface Hydrolysis of PET: Effect of Structural Diversity on Kinetic Properties of Cutinases from Thermobifida. Macromolecules 44 (12), 4632–4640. 10.1021/ma200949p

[B29] Herrero Acero E. Ribitsch D. Dellacher A. Zitzenbacher S. Marold A. Steinkellner G. (2013). Surface Engineering of a Cutinase fromThermobifida Cellulosilyticafor Improved Polyester Hydrolysis. Biotechnol. Bioeng. 110 (10), 2581–2590. 10.1002/bit.24930 23592055

[B30] Hetemi D. Pinson J. (2017). Surface Functionalisation of Polymers. Chem. Soc. Rev. 46 (Issue 19), 5701–5713. 10.1039/c7cs00150a 28766657

[B31] Heumann S. Eberl A. Fischer-Colbrie G. Pobeheim H. Kaufmann F. Ribitsch D. (2009). A Novel Aryl Acylamidase fromNocardia Farcinicahydrolyses Polyamide. Biotechnol. Bioeng. 102 (4), 1003–1011. 10.1002/bit.22139 18942140

[B32] Hoesl M. G. Budisa N. (2012). Recent Advances in Genetic Code Engineering in *Escherichia coli* . Curr. Opin. Biotechnol. 23 (5), 751–757. Elsevier Current Trends. 10.1016/j.copbio.2011.12.027 22237016

[B33] Hoesl M. G. Acevedo-Rocha C. G. Nehring S. Royter M. Wolschner C. Wiltschi B. (2011). Lipase Congeners Designed by Genetic Code Engineering. ChemCatChem 3 (1), 213–221. 10.1002/cctc.201000253

[B34] Kawabata T. Oda M. Kawai F. (2017). Mutational Analysis of Cutinase-like Enzyme, Cut190, Based on the 3D Docking Structure with Model Compounds of Polyethylene Terephthalate. J. Biosci. Bioeng. 124 (1), 28–35. 10.1016/J.JBIOSC.2017.02.007 28259444

[B35] Kawai F. Oda M. Tamashiro T. Waku T. Tanaka N. Yamamoto M. (2014). A Novel Ca2+-Activated, Thermostabilized Polyesterase Capable of Hydrolyzing Polyethylene Terephthalate from *Saccharomonospora Viridis* AHK190. Appl. Microbiol. Biotechnol. 98 (24), 10053–10064. 10.1007/S00253-014-5860-Y/TABLES/3 24929560

[B36] Kawai F. Kawabata T. Oda M. (2019). Current Knowledge on Enzymatic PET Degradation and its Possible Application to Waste Stream Management and Other fields. Appl. Microbiol. Biotechnol. 103 (Issue 11), 4253–4268. Springer Verlag. 10.1007/s00253-019-09717-y 30957199 PMC6505623

[B37] Kleeberg I. Welzel K. VandenHeuvel J. Müller R.-J. Deckwer W.-D. (2005). Characterization of a New Extracellular Hydrolase from Thermobifida Fusca Degrading Aliphatic−Aromatic Copolyesters. Biomacromolecules 6 (1), 262–270. 10.1021/bm049582t 15638529

[B38] Link A. J. Tirrell D. A. (2003). Non-Canonical Amino Acids in Protein Engineering. Curr. Opin. Biotechnol. 14, 603–609. 10.1016/j.copbio.2003.10.011 14662389

[B39] Marten E. Müller R.-J. Deckwer W.-D. (2003). Studies on the Enzymatic Hydrolysis of Polyesters I. Low Molecular Mass Model Esters and Aliphatic Polyesters. Polym. Degrad. Stab. 80 (3), 485–501. 10.1016/S0141-3910(03)00032-6

[B40] Moroder L. Budisa N. (2010). Synthetic Biology of Protein Folding. Chem. Eur. J. Chem. Phys. 11 (6), 1181–1187. 10.1002/cphc.201000035 20391526

[B41] Mozetič M. (2019). Surface Modification to Improve Properties of Materials. Materials 12 (3), 441. 10.3390/ma12030441 PMC6384733 30709009

[B42] Ngo J. T. Tirrell D. A. (2011). Noncanonical Amino Acids in the Interrogation of Cellular Protein Synthesis. Acc. Chem. Res. 44 (9), 677–685. 10.1021/ar200144y 21815659 PMC3178009

[B43] Okada M. Tachikawa K. Aoi K. (1997). Biodegradable Polymers Based on Renewable Resources. II. Synthesis and Biodegradability of Polyesters Containing Furan Rings. J. Polym. Sci. A. Polym. Chem. 35 (13), 2729–2737. 10.1002/(sici)1099-0518(19970930)35:13<2729:aid-pola18>3.0.co;2-d

[B44] Parsons J. F. Xiao G. Gilliland G. L. Armstrong R. N. (1998). Enzymes Harboring Unnatural Amino Acids: Mechanistic and Structural Analysis of the Enhanced Catalytic Activity of a Glutathione Transferase Containing 5-Fluorotryptophan,. Biochemistry 37 (18), 6286–6294. 10.1021/bi980219e 9572843

[B45] Pellis A. Acero E. H. Weber H. Obersriebnig M. Breinbauer R. Srebotnik E. (2015). Biocatalyzed Approach for the Surface Functionalization of poly(L‐lactic Acid) Films Using Hydrolytic Enzymes. Biotechnol. J. 10 (11), 1739–1749. 10.1002/biot.201500074 25963883

[B46] Perz V. Baumschlager A. Bleymaier K. Zitzenbacher S. Hromic A. Steinkellner G. (2016a). Hydrolysis of Synthetic Polyesters byClostridium Botulinumesterases. Biotechnol. Bioeng. 113 (5), 1024–1034. 10.1002/bit.25874 26524601

[B47] Perz V. Hromic A. Baumschlager A. Steinkellner G. Pavkov-Keller T. Gruber K. (2016b). An Esterase from Anaerobic *Clostridium Hathewayi* Can Hydrolyze Aliphatic-Aromatic Polyesters. Environ. Sci. Technol. 50 (6), 2899–2907. 10.1021/acs.est.5b04346 26878094

[B48] Ribitsch D. Heumann S. Trotscha E. Herrero Acero E. Greimel K. Leber R. (2011). Hydrolysis of Polyethyleneterephthalate by P-Nitrobenzylesterase from *Bacillus Subtilis* . Biotechnol. Prog. 27 (4), 951–960. 10.1002/btpr.610 21574267

[B49] Ribitsch D. Acero E. H. Greimel K. Eiteljoerg I. Trotscha E. Freddi G. (2012a). Characterization of a New Cutinase fromThermobifida Albafor PET-Surface Hydrolysis. Biocatal. Biotransform. 30 (1), 2–9. 10.3109/10242422.2012.644435

[B50] Ribitsch D. Herrero Acero E. Greimel K. Dellacher A. Zitzenbacher S. Marold A. (2012b). A New Esterase from *Thermobifida Halotolerans* Hydrolyses Polyethylene Terephthalate (PET) and Polylactic Acid (PLA). Polymers 4 (1), 617–629. 10.3390/polym4010617

[B51] Ribitsch D. Yebra A. O. Zitzenbacher S. Wu J. Nowitsch S. Steinkellner G. (2013). Fusion of Binding Domains to *Thermobifida Cellulosilytica* Cutinase to Tune Sorption Characteristics and Enhancing PET Hydrolysis. Biomacromolecules 14 (6), 1769–1776. 10.1021/bm400140u 23718548

[B52] Ribitsch D. Herrero Acero E. Przylucka A. Zitzenbacher S. Marold A. Gamerith C. (2015). Enhanced Cutinase-Catalyzed Hydrolysis of Polyethylene Terephthalate by Covalent Fusion to Hydrophobins. Appl. Environ. Microbiol. 81 (11), 3586–3592. 10.1128/AEM.04111-14 25795674 PMC4421044

[B53] Roth C. Wei R. Oeser T. Then J. Föllner C. Zimmermann W. (2014). Structural and Functional Studies on a Thermostable Polyethylene Terephthalate Degrading Hydrolase from *Thermobifida Fusca* . Appl. Microbiol. Biotechnol. 98 (18), 7815–7823. 10.1007/s00253-014-5672-0 24728714

[B54] Royter M. Schmidt M. Elend C. Höbenreich H. Schäfer T. Bornscheuer U. T. (2009). Thermostable Lipases from the Extreme Thermophilic Anaerobic Bacteria *Thermoanaerobacter Thermohydrosulfuricus* SOL1 and Caldanaerobacter Subterraneus Subsp. Tengcongensis. Extremophiles 13 (5), 769–783. 10.1007/s00792-009-0265-z 19579003 PMC2757599

[B55] Seidman C. E. Struhl K. Sheen J. Jessen T. (2001). “Introduction of Plasmid DNA into Cells,” in Current Protocols in Molecular Biology (John Wiley & Sons), Chapter 1, 1.8.1–1.8.10. 10.1002/0471142727.mb0108s37 18265047

[B56] Shirke A. N. Basore D. Butterfoss G. L. Bonneau R. Bystroff C. Gross R. A. (2016). Toward Rational Thermostabilization of Aspergillus oryzae Cutinase: Insights into Catalytic and Structural Stability. Proteins 84 (1), 60–72. 10.1002/prot.24955 26522152 PMC4715774

[B57] Son H. F. Cho I. J. Joo S. Seo H. Sagong H.-Y. Choi S. Y. (2019). Rational Protein Engineering of Thermo-Stable PETase from *Ideonella Sakaiensis* for Highly Efficient PET Degradation. ACS Catal. 9 (4), 3519–3526. 10.1021/acscatal.9b00568

[B58] Steiner K. Schwab H. (2012). Recent Advances in Rational Approaches for Enzyme Engineering. Comput. Struct. Biotechnol. J. 2 (3), e201209010. 10.5936/csbj.201209010 24688651 PMC3962183

[B59] Steinkellner G. Rader R. Thallinger G. G. Kratky C. Gruber K. (2009). VASCo: Computation and Visualization of Annotated Protein Surface Contacts. BMC Bioinf. 10 (1), 32. 10.1186/1471-2105-10-32 PMC2649047 19166624

[B60] Sulaiman S. Yamato S. Kanaya E. Kim J.-J. Koga Y. Takano K. (2012). Isolation of a Novel Cutinase Homolog with Polyethylene Terephthalate-Degrading Activity from Leaf-branch Compost by Using a Metagenomic Approach. Appl. Environ. Microbiol. 78 (5), 1556–1562. 10.1128/AEM.06725-11 22194294 PMC3294458

[B61] Thomson J. Ratnaparkhi G. S. Varadarajan R. Sturtevant J. M. Richards F. M. Richards F. M. (1994). Thermodynamic and Structural Consequences of Changing a Sulfur Atom to a Methylene Group in the M13Nle Mutation in Ribonuclease-S. Biochemistry 33 (28), 8587–8593. 10.1021/bi00194a025 8031793

[B62] Thumarat U. Nakamura R. Kawabata T. Suzuki H. Kawai F. (2012). Biochemical and Genetic Analysis of a Cutinase-type Polyesterase from a Thermophilic *Thermobifida alba* AHK119. Appl. Microbiol. Biotechnol. 95 (2), 419–430. 10.1007/s00253-011-3781-6 22183084

[B63] Voloshchuk N. Montclare J. K. (2009). Incorporation of Unnatural Amino Acids for Synthetic Biology. Mol. Biosyst. 6 (Issue 1), 65–80. 10.1039/b909200p 20024068

[B64] Votchitseva Y. A. Efremenko E. N. Varfolomeyev S. D. (2006). Insertion of an Unnatural Amino Acid into the Protein Structure: Preparation and Properties of 3-Fluorotyrosine-Containing Organophosphate Hydrolase. Russ. Chem. Bull. 55 (2), 369–374. 10.1007/s11172-006-0262-7

[B65] Wallace P. W. Haernvall K. Ribitsch D. Zitzenbacher S. Schittmayer M. Steinkellner G. (2017). PpEst Is a Novel PBAT Degrading Polyesterase Identified by Proteomic Screening of *Pseudomonas pseudoalcaligenes* . Appl. Microbiol. Biotechnol. 101 (6), 2291–2303. 10.1007/s00253-016-7992-8 27872998 PMC5320007

[B66] Wei R. Zimmermann W. (2017). Microbial Enzymes for the Recycling of Recalcitrant Petroleum‐based Plastics: How Far Are We? Microb. Biotechnol. 10 (Issue 6), 1308–1322. 10.1111/1751-7915.12710 28371373 PMC5658625

[B67] Weinberger S. Haernvall K. Scaini D. Ghazaryan G. Zumstein M. T. Sander M. (2017). Enzymatic Surface Hydrolysis of Poly(ethylene Furanoate) Thin Films of Various Crystallinities. Green. Chem. 19 (22), 5381–5384. 10.1039/c7gc02905e

[B68] Wiltschi B. Cernava T. Dennig A. Galindo Casas M. Geier M. Gruber S. (2020). Enzymes Revolutionize the Bioproduction of Value-Added Compounds: From Enzyme Discovery to Special Applications. Biotechnol. Adv. 40, 107520. 10.1016/J.BIOTECHADV.2020.107520 31981600

[B69] Wolschner C. Giese A. Kretzschmar H. A. Huber R. Moroder L. Budisa N. (2009). Design of Anti- and Pro-aggregation Variants to Assess the Effects of Methionine Oxidation in Human Prion Protein. Proc. Natl. Acad. Sci. 106 (19), 7756–7761. 10.1073/PNAS.0902688106 19416900 PMC2674404

[B70] Xie Q. Sun H. Xie G. Zhou J. (1995). An Iterative Method for Calculation of Group Electronegativities. J. Chem. Inf. Comput. Sci. 35 (1), 106–109. 10.1021/ci00023a015

[B71] Yoshida S. Hiraga K. Takehana T. Taniguchi I. Yamaji H. Maeda Y. (2016). A Bacterium that Degrades and Assimilates Poly(ethylene Terephthalate). Science 351 (6278), 1196–1199. 10.1126/science.aad6359 26965627

[B72] Zheng S. Kwon I. (2012). Manipulation of Enzyme Properties by Noncanonical Amino Acid Incorporation. Biotechnol. J. 7 (1), 47–60. 10.1002/biot.201100267 22121038

