# Supplementary material for: Residue-Specific Incorporation of the Non-Canonical Amino Acid Norleucine Improves Lipase Activity on Synthetic Polyesters
Source: Front Bioeng Biotechnol. 2022 Jan 26;10:769830. doi: 10.3389/fbioe.2022.769830 (PMC8826565; doi:10.3389/fbioe.2022.769830)
Supplement: Supplementary file 1 [file DataSheet1.docx]

Residue-specific incorporation of the non-canonical amino acid norleucine improves lipase activity on synthetic polyesters

**Karolina Haernvall^1,^** ^⸸^**, Patrik Fladischer^1,2,^** ^⸸^**, Heidemarie Schoeffmann^1^, Sabine Zitzenbacher^1^, Tea Pavkov-Keller^1,4,5,6^, Karl Gruber^4,5,6^, Michael Schick^7^, Motonori Yamamoto7, Andreas Kuenkel^7^, Doris Ribitsch ^1,c3,*^, Georg M. Guebitz^1,3^ and Birgit Wiltschi^1,2,5^**

^1^acib - Austrian Centre of Industrial Biotechnology, Graz, Austria

^2^ Institute of Molecular Biotechnology, Graz University of Technology, Graz, Austria

^3^Institute for Environmental Biotechnology, University of Natural Resources and Life Sciences, Vienna, Tulln an der Donau, Austria

^4^Institute of Molecular Biosciences, University of Graz, Graz, Austria

^5^BioTechMed-Graz, Graz, Austria

^6^Field of Excellence BioHealth – University of Graz, Austria

^7^BASF SE, Ludwigshafen am Rhein, Germany

*** Correspondence:**Doris Ribitsch, [doris.ribitsch@boku.ac.at](mailto:doris.ribitsch@boku.ac.at)


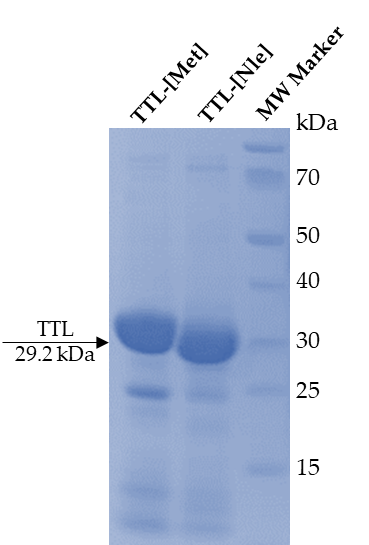


**Supplementary Figure S1.** Purified TTL variants separated on a 10% SDS-PA gel. The size of the relevant molecular weight marker bands (MW marker) is indicated on the right.


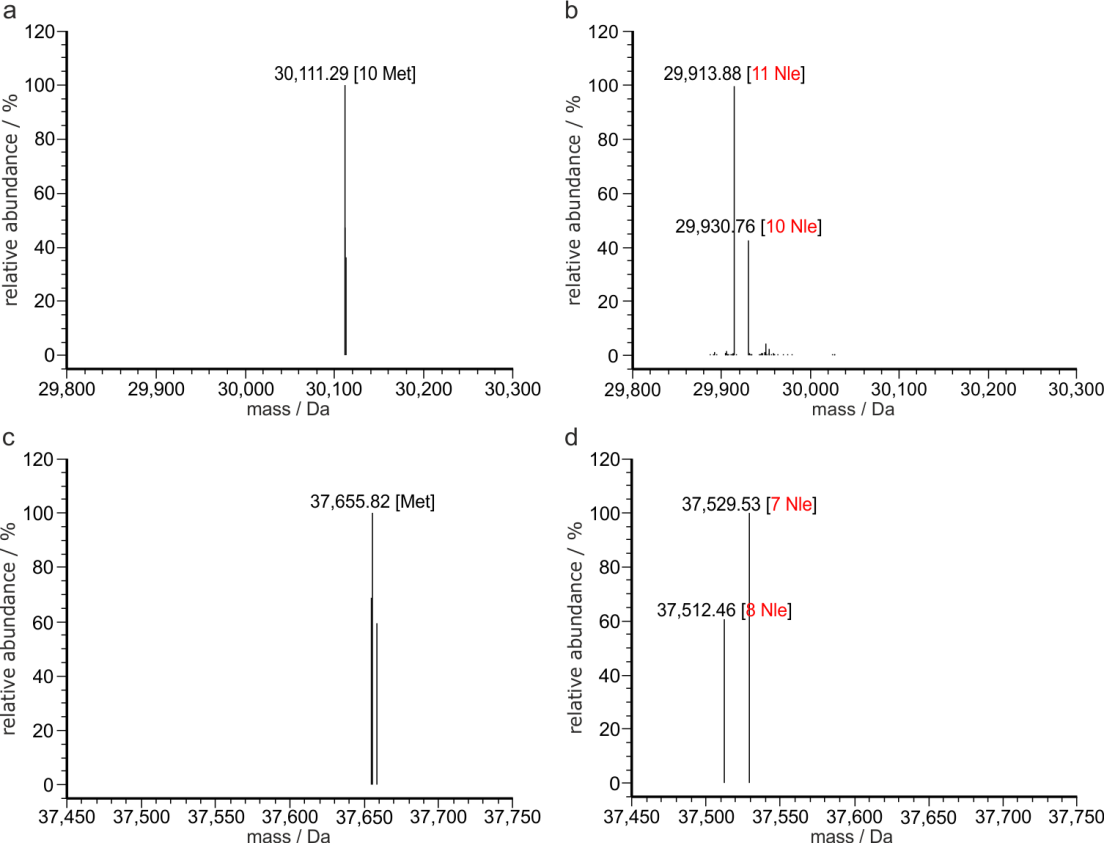


**Supplementary Figure S2.** Intact protein mass spectra of the TTL[Nle] variants. Monoisotopic LC-ESI MS-spectra of TTL[Met] (a), TTL[Nle] (b) are shown. The main peaks are labeled with the experimentally determined masses; the number of incorporated ncAA residues is shown in brackets.


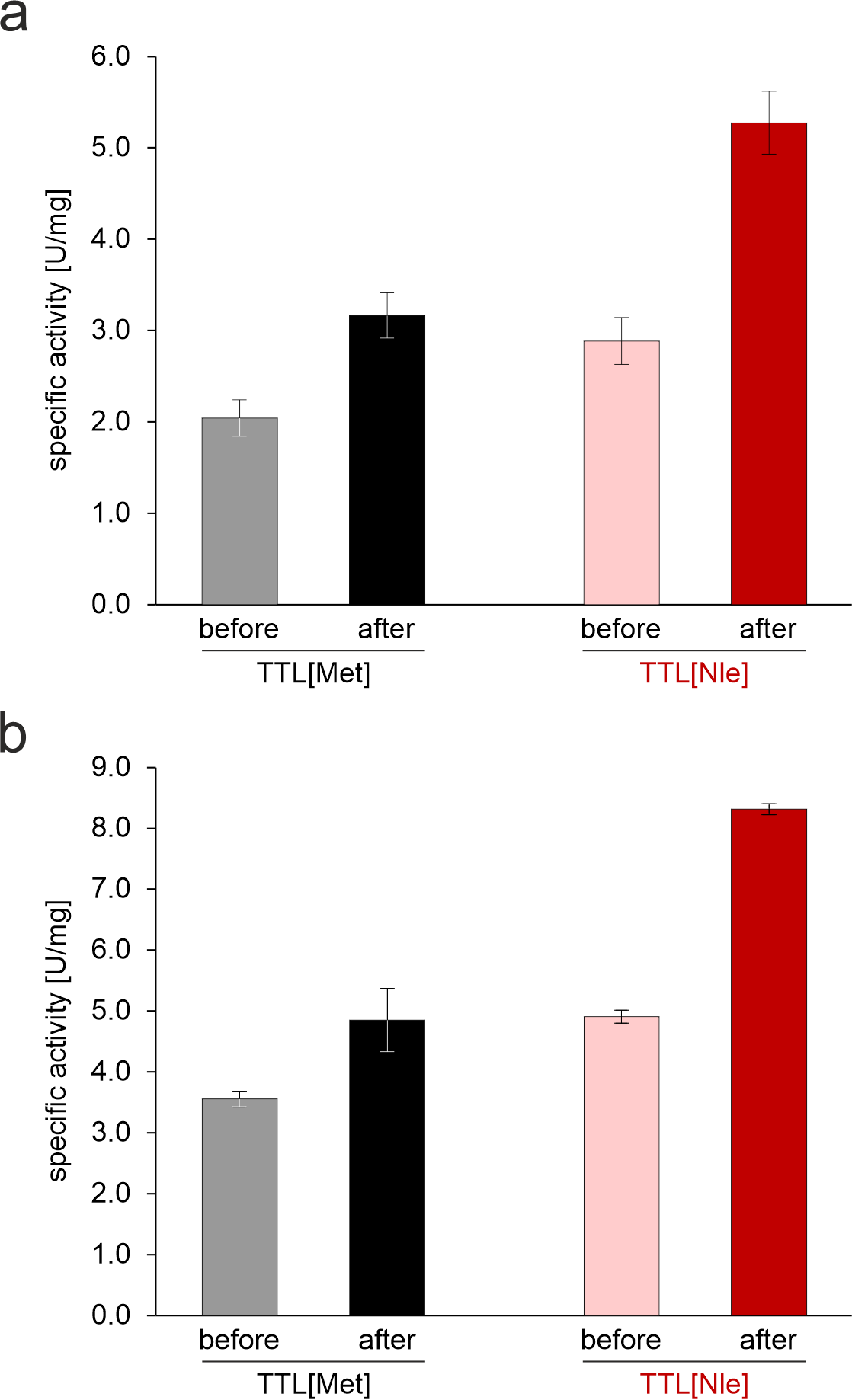


**Supplementary Figure S3.** TTL[Met] and TTL[Nle] variants were incubation with PET films for 24 h at 70 °C in 100 mM Tris/Cl pH 7. We assessed the esterase activity of TTL[Met] and TTL[Nle] before (before) the enzymes were incubated with PET films and afterwards (after). The esterase activity of the enzymes was determined in triplicates using *para*-nitrophenylacetate (a) and *para*-nitrophenylbutyrate (b) as the substrates. One esterase unit (U) is defined as the amount of enzyme that liberates 1 μmol *para*-nitrophenol per min under the assay conditions described.
